# The mechanism of complex formation between calmodulin and voltage gated calcium channels revealed by molecular dynamics

**DOI:** 10.1371/journal.pone.0258112

**Published:** 2021-10-05

**Authors:** Shivani Yaduvanshi, Rya Ero, Veerendra Kumar

**Affiliations:** 1 Amity Institute of Molecular Medicine and Stem Cell Research (AIMMSCR), Amity University Noida, Noida, Uttar Pradesh, India; 2 Institute of Molecular and Cell Biology, University of Tartu, Tartu, Estonia; Russian Academy of Medical Sciences, RUSSIAN FEDERATION

## Abstract

Calmodulin, a ubiquitous eukaryotic calcium sensor responsible for the regulation of many fundamental cellular processes, is a highly flexible protein and exhibits an unusually wide range of conformations. Furthermore, CaM is known to interact with more than 300 cellular targets. Molecular dynamics (MD) simulation trajectories suggest that EF-hand loops show different magnitudes of flexibility. Therefore, the four EF-hand motifs have different affinities for Ca^2+^ ions, which enables CaM to function on wide range of Ca^2+^ ion concentrations. EF-hand loops are 2–3 times more flexible in apo CaM whereas least flexible in Ca^2+^/CaM-IQ motif complexes. We report a unique intermediate conformation of Ca^2+^/CaM while transitioning from extended to compact form. We also report the complex formation process between Ca^2+^/CaM and IQ CaM-binding motifs. Our results showed how IQ motif recognise its binding site on the CaM and how CaM transforms from extended to compact form upon binding to IQ motif.

## Introduction

Calmodulin (Calcium-modulated protein, a.k.a. CaM) is a “dumbbell” shaped protein ubiquitously present in eukaryotes that mediates calcium-dependent signalling. CaM can bind four calcium ions (Ca^2+^) in its N- and C-lobes (two ions *per* lobe) and thereby regulate numerous Ca^2+^-dependent pathways [1–3]. Both N- and C-lobes consist of two highly conserved canonical EF-hand motifs [4]. In each EF-hand, two α helices are connected by a 12-residue long acidic loop (helix-turn-helix). Ca^2+^ binding rearranges the helices in EF-hand and exposes large phenylalanine (Phe) and methionine (Met) rich hydrophobic clefts for target binding [5–7]. In the absence of Ca^2+^, the α-helices are parallel to one another (called “closed conformation”), whereas in the presence of Ca^2+^, the α-helices are perpendicular to one another (called “open conformation”) [8, 9]. Interestingly, the two lobes have different affinities for Ca^2+^ and therefore also for target binding [10]. Previous studies have revealed that the four Ca^2+^-binding sites (EF- hands) differ in their ability to bind Ca^2+^ ions. For example, Ca^2+^ ions bind the C-lobe (*K*_d_∼10^−6^ M) with 10-fold higher affinity than the N-lobe (*K*_d_∼10^−5^ M) [11, 12]. Therefore, CaM can sense a broad range of Ca^2+^ signals and convert Ca^2+^ influx into cellular signals by binding to more than 300 partners.

The two lobes of CaM are connected by a highly flexible central helix in a *trans* orientation [13–15]. The linker is largely exposed to the solvent and is involved in target binding [7, 9]. A fragment of the linker (from arginine-74 to glutamate-83) can unwind to various degrees so that the two lobes of CaM can re-orient and wrap around the target peptide [14, 16]. Alternatively, target can bind to one of the lobes with no bending taking place in the central helix [17].

Voltage gated calcium channels (VGCC) are multimeric proteins found in the membrane of excitable cells [18–22]. In response to membrane depolarization, VGCCs mediate Ca^2+^ influx that initiates several physiological events. There are 10 subfamilies of VGCCs critically important for brain, heart, and muscle functions [23–25]. VGCCs are divided into L-type (CaV1.1, CaV1.2, CaV1.3, and CaV1.4), P/Q-type (CaV2.1), N-type (CaV2.2), and R-type (CaV2.3) [13, 26]. VGCCs are regulated by CaM through their C-terminal IQ motifs (I/L/V) QXXXRXXXX(R/K) [27–30]. CaM controls the calcium influx by calcium-dependent inactivation (CDI) and calcium-dependent facilitation (CDF) [31]. Numerous studies suggest that IQ domain mutations in full-length channels can eliminate CDI and CDF [32–34]. Thus, it will be interesting to know how CaM regulates various ion channels through interaction with their IQ motifs.

Despite extensive studies, many details about CaM are still not fully understood. For example, how CaM can sense a broad range of Ca^2+^ concentrations and adopt a multitude of conformations? It will be interesting to find out how the IQ motifs interact with the CaM molecule. In this study, we carried out classical molecular dynamic simulations of various Ca^2+^/CaM and VGCC IQ motif complexes. We analyse the trajectory to understand the dynamics of EF-hand helices and the central helix of CaM. We also report the conformational landscape of CaM transforming from extended to compact conformations during complex formation.

## Materials and methods

Molecular dynamics (MD) simulations were performed for apo CaM (Protein Data Bank ID: 1CFD), Ca^2+^/CaM (PDB ID: 1CLL), Ca^2+^/CaM (PDB ID: 1PRW), and Ca^2+^/CaM-IQ motif complexes. The following structures of CaM and IQ motifs from VGCC complexes were used for MD simulations: CaV1.2 IQ (2BE6), CaV1.1 IQ (2VAY), CaV2.3 IQ (3BXL), CaV2.2 IQ (3DVJ), and CaV2.1 IQ (3DVM). All simulations were performed using Gromacs 2020.2 for 50 nanoseconds (ns) using CHARMM36 force field [35]. Simulation inputs were built using CHARMM-GUI web [36, 37]. Protein molecules were placed in a cubic box located 10 Å (10^−10^ meter) from box boundaries. Solvent water was filled with tip3p model around the protein molecules [38]. Four Ca^2+^ ions were present in CaM for all MD simulations except for apo CaM. The simulation time was selected based on previous literature [39] and observing the RMSD profile. As shown in Fig 2, RMSD stabilizes after around 45 sec and no significant changes occur after that. While longer MD simulations have been reported [40, 41], we observed that the dynamic behaviour of CaM occurred within 50 ns. Therefore, to optimize the computing, we limited the simulation to 50 ns for all complexes. Protein molecules were neutralized with 0.15M NaCl. Simulations were performed with periodic boundary conditions. The energy minimization steps comprising gradual reduction of side chain and backbone restraints were carried out for 250 picoseconds (ps). Water equilibration around the protein molecule was performed under NVT for 125 ps followed by 125 ps NPT ensembles at 303 K. The production run was performed at 303 K in the NPT ensemble for 50 ns. The time step was 2 femtosecond (fs) and the trajectory was saved every 10 ps. Temperature was maintained using velocity scaling. Bond lengths were constrained with the LINCS algorithm. The pressure was controlled by isotropic coupling using Parrinello-Rahman barostat. A Verlet scheme was used for van der Waals and Particle Mesh Ewald electrostatics (PME) interactions within 1.2 nm. Van der Waals interactions were switched above 1.0 nm. The progress of the simulations was monitored by RMSD profiles.

MD results were analysed in Gromacs 2020.2 package utilities. MD trajectories were concatenated by using trjcat utility of Gromacs package. The multiple chains of protein molecules were clustered using trjconv utility of Gromacs package with an option for periodic boundary conditions of clusters. This option fixes the structure broken by the periodic boundary.

## Results

**Fig 1:**
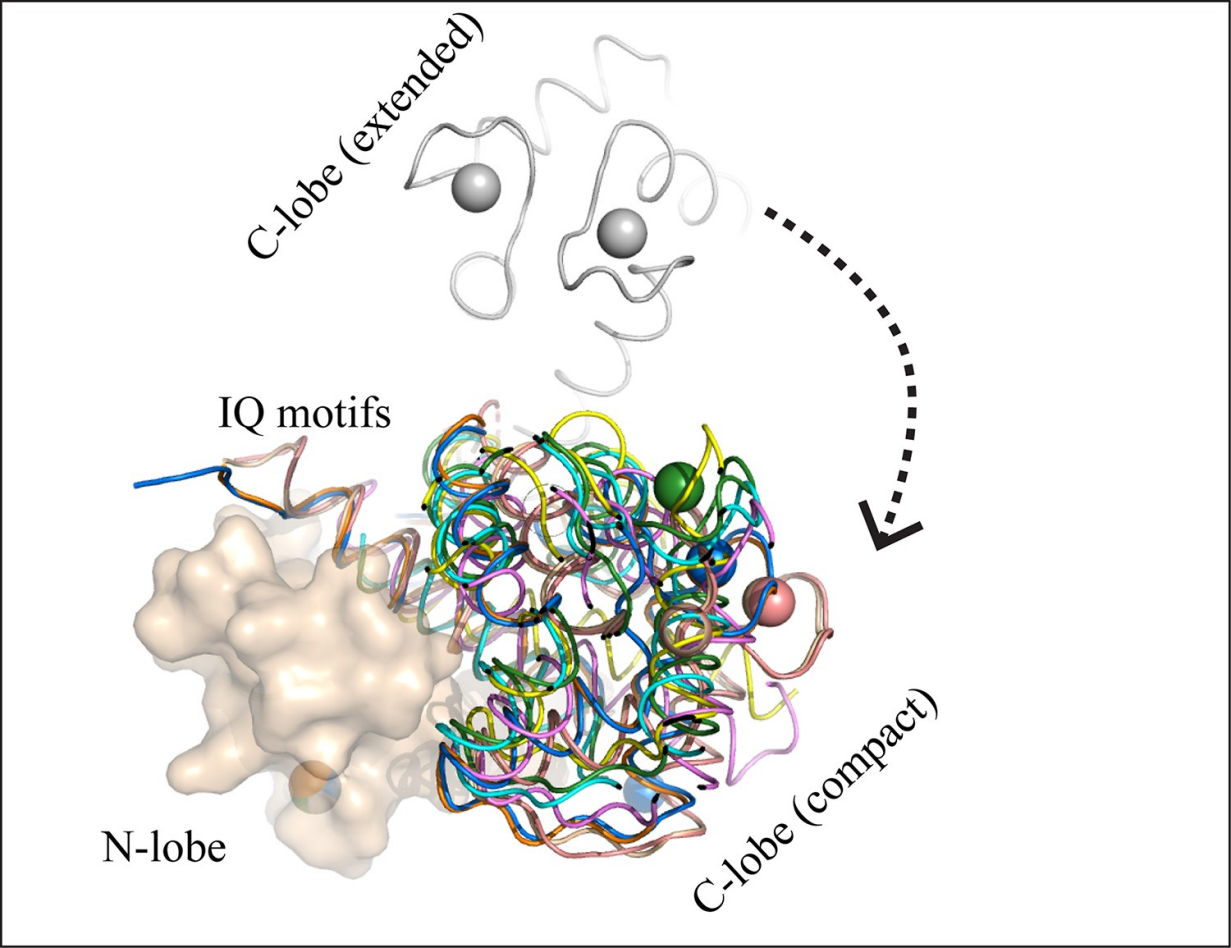
Comparison of the compact and the extended conformations of CaM. The CaM exist in extended form as well as in compact form. In the extended form, the N- and C-lobes are in *trans* configuration whereas in the compact form, the lobes are in *cis* configuration. CaM central helix bends to bring the C- and N-lobes close to one another. The C-lobe rotates around the axis to adopt the compact conformation. CaM complexes are coloured as follows: extended Ca^2+^/CaM (1CLL, grey), Ca^2+^/CaM-Cav1.1IQ (2VAY, yellow), Ca^2+^/CaM-Cav1.2IQ (1BE6, violet), Ca^2+^/CaM-Cav2.1IQ (3BXK, cyan), Ca^2+^/CaM-Cav2.1IQ (3DVM, wheat), Ca^2+^/CaM-Cav2.2IQ (3DVE, marine), Ca^2+^/CaM-Cav2.2IQ (3DVJ, orange), Ca^2+^/CaM-Cav2.3IQ (3BXL, forest), and Ca^2+^/CaM-Cav2.3IQ (3DVK, salmon). Ca^2+^ are shown in spheres.

Ca^2+^/CaM adopts either extended or compact form in the solution. In all known Ca^2+^-CaM/VGCC IQ complexes, CaM is found in compact conformation (Fig 1). However, in all these compact CaM structures relative position of the two lobes varies. CaM central helix is observed to bend to varying degrees in the reported CaM/VGCC IQ complexes placing the two lobes in different positions. C-lobes rotate between 140–150° to adopt compact form in various CaM/VGCC IQ complexes. Least rotation occurs (133.85°) in Ca^2+^/CaM-IQCav1.1 complex structure (2VAY) (Fig 1 and Table 1). Whereas in apo and extended Ca^2+^/CaM, the two lobes are in *trans* conformations, but in all compact CaM, lobes are in *cis* conformation because of the bending of central helix (Fig 1).

**Table 1:** Rotation angles of CaM C-lobes in various reported compact Ca^2+^/CaM-IQ motif complexes structures.

Complex	Rotation angel (°)
CaM-IQCav1.1 (2VAY)	133.85
CaM-IQCaV1.2 (2BE6)	143.0
CaM-IQCav2.1 (3DVM)	149.0
CaM-IQCav2.2 (3DVJ)	143.2
CaM-IQCav2.3 (3DVK)	149.54
Compact CaM (1PRW)	142.0

### Dynamic behaviour of Ca^2+^/CaM-IQ complexes

**Fig 2:**
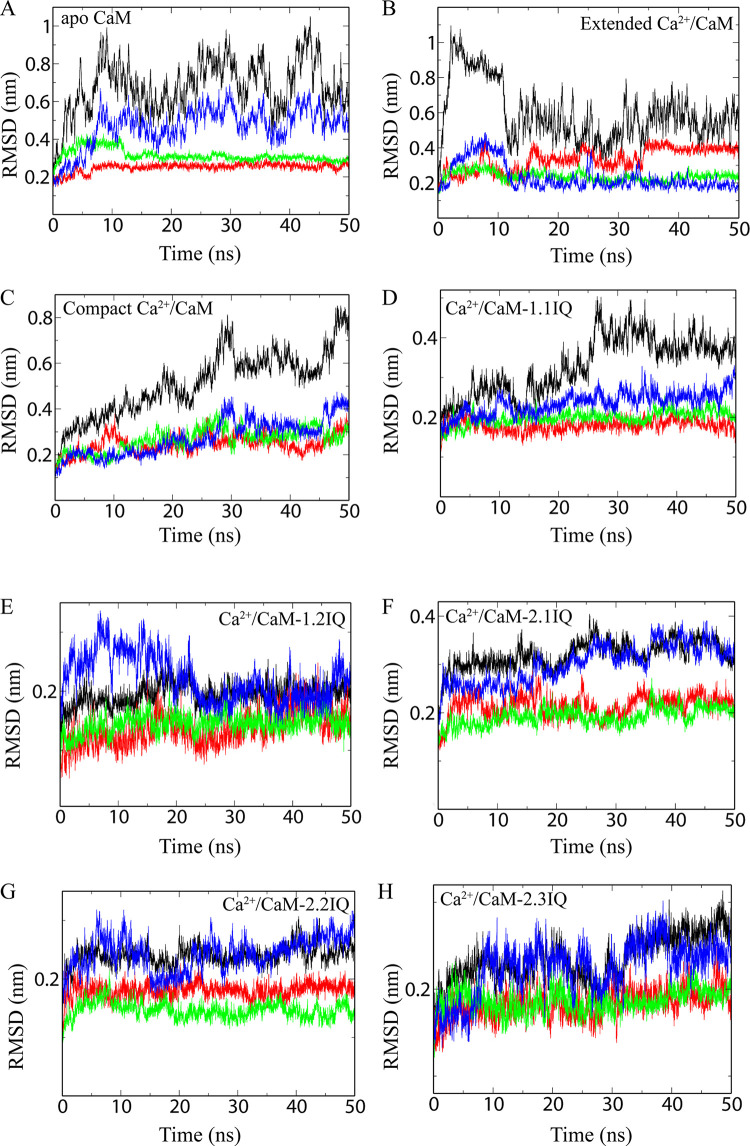
Root-mean-square deviation (RMSD) of CaM complexes. **(A)** apo CaM (1CFD), **(B)** extended Ca^2+^/CaM (1CLL), **(C)** compact Ca^2+^/CaM (1PRW), **(D)** Ca^2+^/CaM-Cav1.1IQ (2VAY), **(E)** Ca^2+^/CaM-Cav1.2IQ (2BE6), **(F)** Ca^2+^/CaM-Cav2.1IQ (3DVM), **(G)** Ca^2+^/CaM-Cav2.2IQ (3DVJ), and **(H)** Ca^2+^/CaM-Cav2.3IQ (3BXL). The RMSD curve of the whole complex (black), N-lobe (red), C-lobe (green), and central helix (blue) are shown.

To assess the dynamic behaviour of the complexes, the time dependent RMSD of all protein atoms was calculated using original complexes as reference. The apo CaM (without Ca^2+^) showed RMSD between 0.5–1.0 nm for up to 45 ns of simulation and thereafter it becomes constant at ~0.6 nm (Fig 2A). The higher RMSD is mainly due to the central linker (Fig 2A). The N- and C-lobes exhibit relatively low RMSD (~0.3 nm). The extended Ca^2+^/CaM (1CLL) exhibited the highest RMSD value (Fig 2B). RMSD increases close to 1.0 nm at the beginning up to 10 ns. Thereafter, RMSD decreases slowly and stabilizes at ~ 0.5 nm for rest of the simulation period. The increase followed by the decrease in RMSD of the extended Ca^2+^/CaM indicates cracking or a local unfolding of the molecule [42]. As discussed below, it is probably due to the unfolding of the linker fragment [43]. The free-Ca^2+^/CaM (1PRW) can also adopt the compact conformation [44]. The RMSD of the free compact CaM is slightly lower (0.2–0.8 nm) than observed for the extended CaM conformation (Fig 2C). Thus, free CaM (apo as well Ca^2+^/CaM) are highly flexible in solution.

The RMSD curves of all analysed Ca^2+^/CaM-IQ complexes are unique (Fig 2D–2H). In contrast to the apo and free Ca^2+^/CaM, most of the Ca^2+^/CaM-IQ complexes are very stable (RMSD 0.2–0.3 nm) in solution. Thus, RMSD analysis corroborates earlier finding that Ca^2+^ and IQ motifs stabilize the CaM in solution [45–47]. The RMSDs of N- and C-lobes of Ca^2+^/CaM-IQ complexes are between 0.1–0.2 nm; and the central helix RMSDs are 0.2–0.35 nm (Fig 2D–2H). In the Ca^2+^/CaM-IQ complexes, the central helix is involved in IQ motif binding and therefore very less flexible in solution compared to free CaM. Curiously, CaM/Cav1.1IQ (2VAY) RMSD curve is slightly different (Fig 2D). Its RMSD increases until 30 ns and then remains constant at 0.4 nm. However, its N-, C-lobes, and linker exhibit similar RMSD (~0.2–0.25 nm) as observed for other Ca^2+^/CaM-IQ complexes. Biochemical studies suggest that CaM interacts with the “pre-IQ” regions of the Cav1.1 IQ motif [48]. Histidine 1532 of IQ motif will increase the flexibility of a loop of CaM resulting in fewer stabilizing interactions. Three out of the four unique residues in Cav1.1 IQ motif do not directly interact with CaM [49]. Thus, the unique interaction behaviour of Cav1.1 IQ motif creates a flexible region in CaM resulting in higher RMSD.

### Dynamic behaviour of calcium binding loops

**Fig 3:**
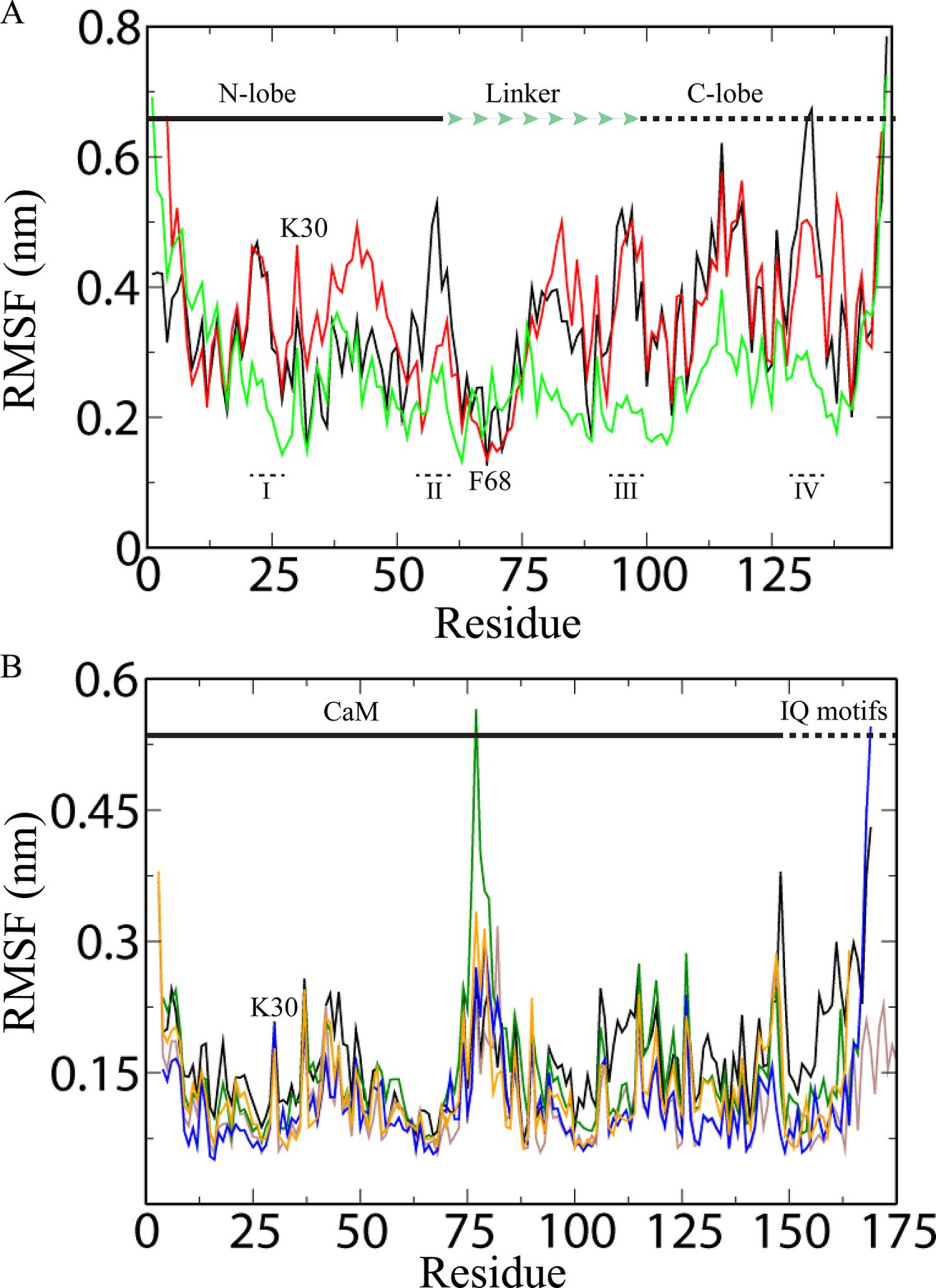
Root-mean-square fluctuations (RMSF) of CaM complexes. **(A)** apo CaM (black), extended Ca^2+^/CaM (red), and compact Ca^2+^/CaM (green); **(B)** Ca^2+^/CaM-Cav1.1IQ (black), Ca^2+^/CaM-Cav1.2IQ (brown), Ca^2+^/CaM-Cav2.1IQ (green), Ca^2+^/CaM-Cav2.2IQ (blue), and Ca^2+^/CaM-Cav2.3IQ (orange). The N- and C-lobes, linker, and IQ motifs are marked. EF hand loops I (D20-T28), loops II (D56-P66), loops III (D93-Y99), and loops IV (I130-E140) are also shown.

**Fig 4:**
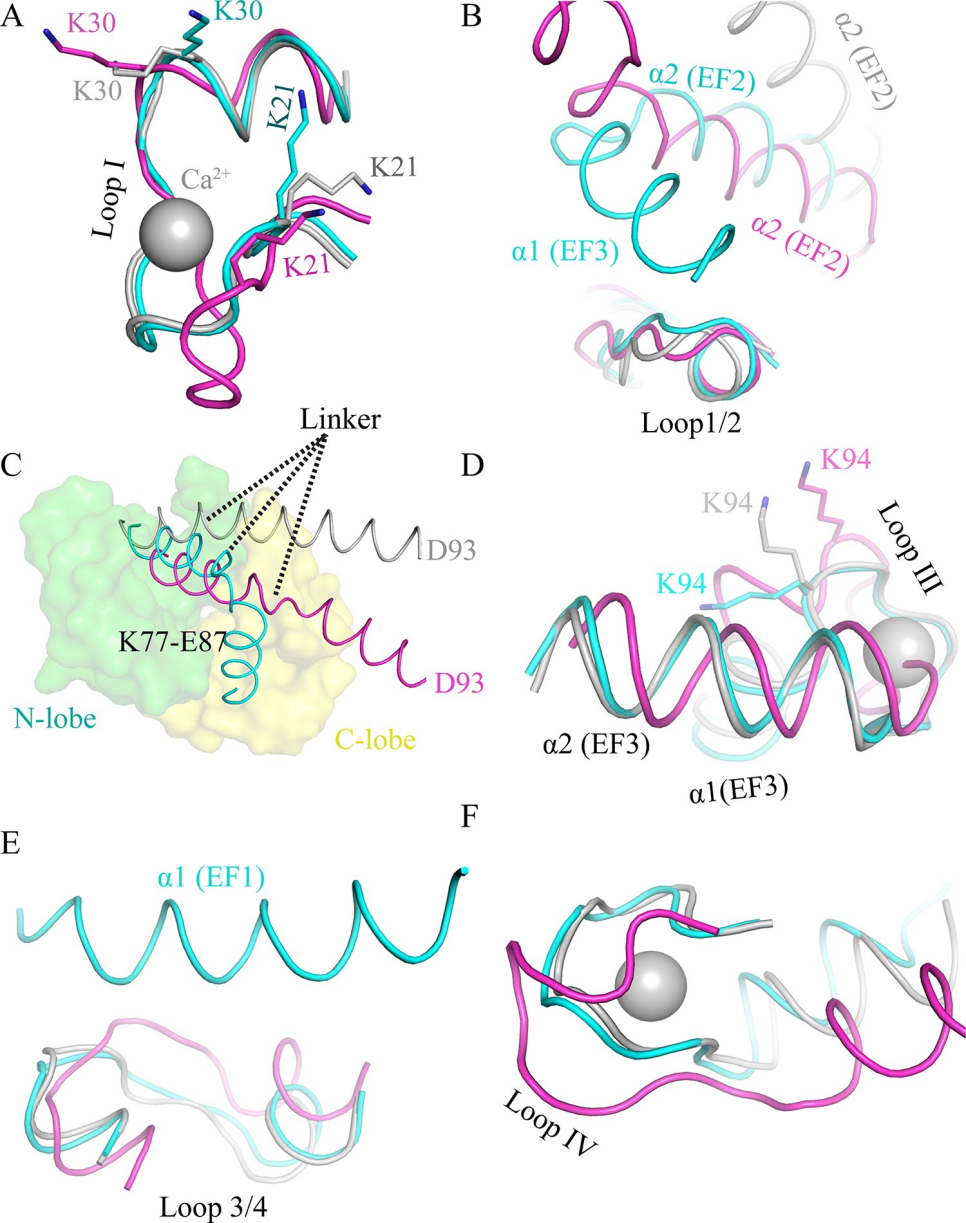
Structural basis of root mean-square fluctuations in various CaM loops regions. **(A)** Loop I (EF1) hand motif is stabilized by the K21 residue in the compact CaM structure. **(B)** Loop 1/2 is stabilized in both the apo CaM and the compact CaM structures by the close proximity of the helices α1 (EF3) and α2 (EF2). **(C)** The central helix is entrapped between the two lobes in the compact CaM structure **(D)** Loop III (EF3) is stabilized by K94 in the compact CaM structure. **(E)** Loop 3/4 exhibits limited movement in compact CaM due to being in close proximity to helix α1 (EF1). **(F)** Loop IV (EF4) is highly flexible in apo CaM due to its unusual conformation. The extended Ca^2+^/CaM, apo CaM, and the compact Ca^2+^/CaM are shown in grey, magenta, and cyan, respectively.

The dynamic behaviour at residue level was estimated by RMSF calculations. RMSF represents the positions of the residues across the simulation trajectory. In apo and extended CaM, the acidic loop I (D20-T28) is flexible in solution (RMSF ~0.45 nm). In contrast, restricted fluctuation is observed in compact CaM (RMSF ~0.25 nm). This loop is involved in Ca^2+^ binding at EF1. In compact Ca^2+^/CaM, K21 is trapped between helix α2 (EF1) and helix α2 (EF3) (Fig 4A) explaining the restricted fluctuation revealed by RMSF (Fig 3A). Also, K30 fluctuates (RMSF ~0.3–0.45 nm) in apo as well as in Ca^2+^/CaM (both compact and extended) because it remains free in all CaM structures (Fig 3A and 3B). Loop1/2 (L39-E47) that connects EF1 and EF2 is on the surface in the extended Ca^2+^/CaM and is highly flexible (RMSF ~ 0.50 nm). However, in compact Ca^2+^/CaM structure, α2 (EF2) and α1 (EF3) position close to this loop restricting its flexibility (RMSF ~0.26 nm) (Figs 3A and 4B). In apo CaM, only α2 (EF2) limits the movement of loop 1/2 (Fig 4B). The EF2 loop II (D56-P66) is less flexible (RMSD 0.2–0.3 nm) compared to EF1 loop in extended conformation. It exhibits even lower RMSD (0.15–0.25 nm) in the compact form. However, this loop is highly flexible in apo CaM (Fig 3A) in agreement with previous studies [42, 50].

The linker residue P66-R74 constitute the α2 helix of EF2 as well as the linker helix. These residues are trapped between N-lobe helices as well as are in proximity to α1 (EF3). Thereby explaining the moderate flexibility (RMSF ~0.23 nm) of this region in all 3 forms of CaM (Fig 3A). Specifically, the residue F68 exhibits the lowest RMSF (~0.15 nm) in both conformation because it is buried deep in the N-lobe (Fig 3A). F68 is involved in the hydrophobic interactions with various targets [51, 52] and F68A mutation is lethal for the cells [53, 54]. Linker residues (K77-E87) are highly flexible (RMSF ~ 0.50 nm) in apo CaM and extended Ca^2+^/CaM. CaM adopts various conformations arising from varying degrees of bending observed in this region (Fig 1 and Table 1). In apo CaM and extended Ca^2+^/CaM, the linker residues form an elongated helix. In the compact form, however, region K77-E87 is sandwiched between the N- and C-lobes (Fig 4C). Furthermore, target peptides bind between the linker regions (P66-R74) and K77-E87 region (discussed below). Therefore, not surprisingly, the region K77-E87 is the least flexible (RMSF ~ 0.20 nm) in the compact form of CaM (Fig 3A).

The temperature factor analysis suggests that the N-lobe is more flexible than the C-lobe, especially in apo CaM (without Ca^2+^) [42]. In the C-lobe, loop III (D93-Y99) is highly flexible in both the apo CaM and the extended Ca^2+^/CaM (RMSF ~0.50 nm) (Fig 3A). This loop is involved in Ca^2+^ coordination at EF3. As in EF1 loop, equivalent lysine (K94) is present in the EF3 loop as well (Fig 4D). Residue K94 anchors the EF3 loop between helices α1 (EF3) and α2 (EF3) in the compact CaM [55, 56]. Therefore, in the compact CaM, this loop shows limited fluctuations in solutions (RMSF 0.20 nm) (Fig 3A). In apo CaM and the extended Ca^2+^/CaM, the loop 3/4 (N111-E120) between EF3 and EF4 is highly flexible (RMSF ~0.57 nm) (Fig 3A). However, in the compact CaM, the flexibility in this region is reduced due to its closeness to α1 (EF1) (Fig 4E). In apo CaM and the extended Ca^2+^/CaM, α1 (EF1) is too far to make any contacts (S1 Fig). Also, the loop IV (I130-E140) is flexible in the extended CaM (RMSF 0.5 nm) but is more rigid in the compact CaM (RMSF 0.22 nm). As with loop II, the loop IV (I130-E140) also shows the highest degree of flexibility in apo CaM (Fig 3A). Interestingly, whereas in extended Ca^2+^/CaM and compact Ca^2+^/CaM, loop IV adopts similar conformation but in apo CaM, loop IV is in totally different conformation (Fig 4F).

Overall, the compact CaM residues are less flexible than apo CaM and the extended CaM. Apo CaM and the extended CaM have similar flexible in region. Notable difference is observed in the region between L39-P66, which contains the EF2-hand motif (Fig 3A). EF-hand motif residues in apo CaM and the extended Ca^2+^/CaM reveal RMSF ~0.35 nm, whereas in compact Ca^2+^/CaM, RMSF is ~0.2 nm (Fig 3A). The loops involved in Ca^2+^ coordination in the four EF-hand motifs, exhibit different extent of flexibility (Table 2). Thus, the four EF-hands are non-identical and have therefore different degrees of affinities for Ca^2+^ which agrees to previous studies [18, 19, 21, 22]. Therefore, CaM can sense a broad range of Ca^2+^ concentration [18, 19, 21, 22]. Further, their flexibility decreases in following order: apo CaM > extended Ca^2+^/CaM >compact Ca^2+^/CaM (Table 2). Thus, Ca^2+^ binding stabilizes the EF-hand motifs [42]. Comparison of flexibility of loops that connect the EF-hand motifs suggest that EF connecting loops are the least flexible in compact Ca^2+^/CaM (Table 2). It is also worth to note that many residues come close to each other by flexible motions of central helix [57]. Hence, the flexibility of individual residues varies during simulation. As discussed below, further reduction in flexibility is observed when compact Ca^2+^/CaM interacts with IQ peptides.

**Table 2:** RMSD of different loops of Ca^2+^ binding region of CaM.

	Apo CaM (nm)	Extended Ca^2+^/CaM (nm)	compact Ca^2+^/CaM (nm)
**Loop I (D20-T28)**	0.45	0.45	0.25
**loop II (D56-P66)**	0.53	0.25	0.2
**loop III (D93-Y99)**	0.51	0.5	0.2
**loop IV (I130-E140)**	0.67	0.55	0.22
**loop 1/2 (L39-E47)**	0.3	0.5	0.3
**loop 2/3 (K77-E87)**	0.4	0.5	0.24
**loop 3/4 (N111-E120)**	0.6	0.6	0.4

### Dynamic behaviour of calcium binding loops of CaM-VGCC IQ complexes

CaM adopts compact conformation upon binding to the IQ motifs of VGCCs [33]. The flexible regions of extended Ca^2+^/CaM become rigid in CaM-IQ complexes. Overall, the residues show very low magnitudes of fluctuation (RMSF < 0.2 nm) (Fig 3B). Particularly, the EF motif residues exhibit very low RMSF (~0.1 nm) because of their involvement in IQ motif binding and Ca^2+^ coordination [58]. Most of the hydrophobic residues are involved in either target or intra-molecular interactions. The linker region R74-D80 reveals higher flexibility (RMSF ~0.35 nm). This region unwinds and bends to make CaM more compact for complex formation. Due to the complete unwinding of linker region, CaM can adopt a variety of conformations guided by targets [58]. The bound IQ peptides lock the two lobes in a specific conformations. Also, the negatively charged residues in Ca^2+^/CaM make strong electrostatic interactions with the positively charged IQ motif residues [59]. Consequently, the N- and C-lobes exhibit very low RMSF values. Taken together, the above analysis revealed that the flexibility of the Ca^2+^ binding loop and the central helix decreases from apo to compact Ca^2+^/CaM. [42].

**Fig 5:**
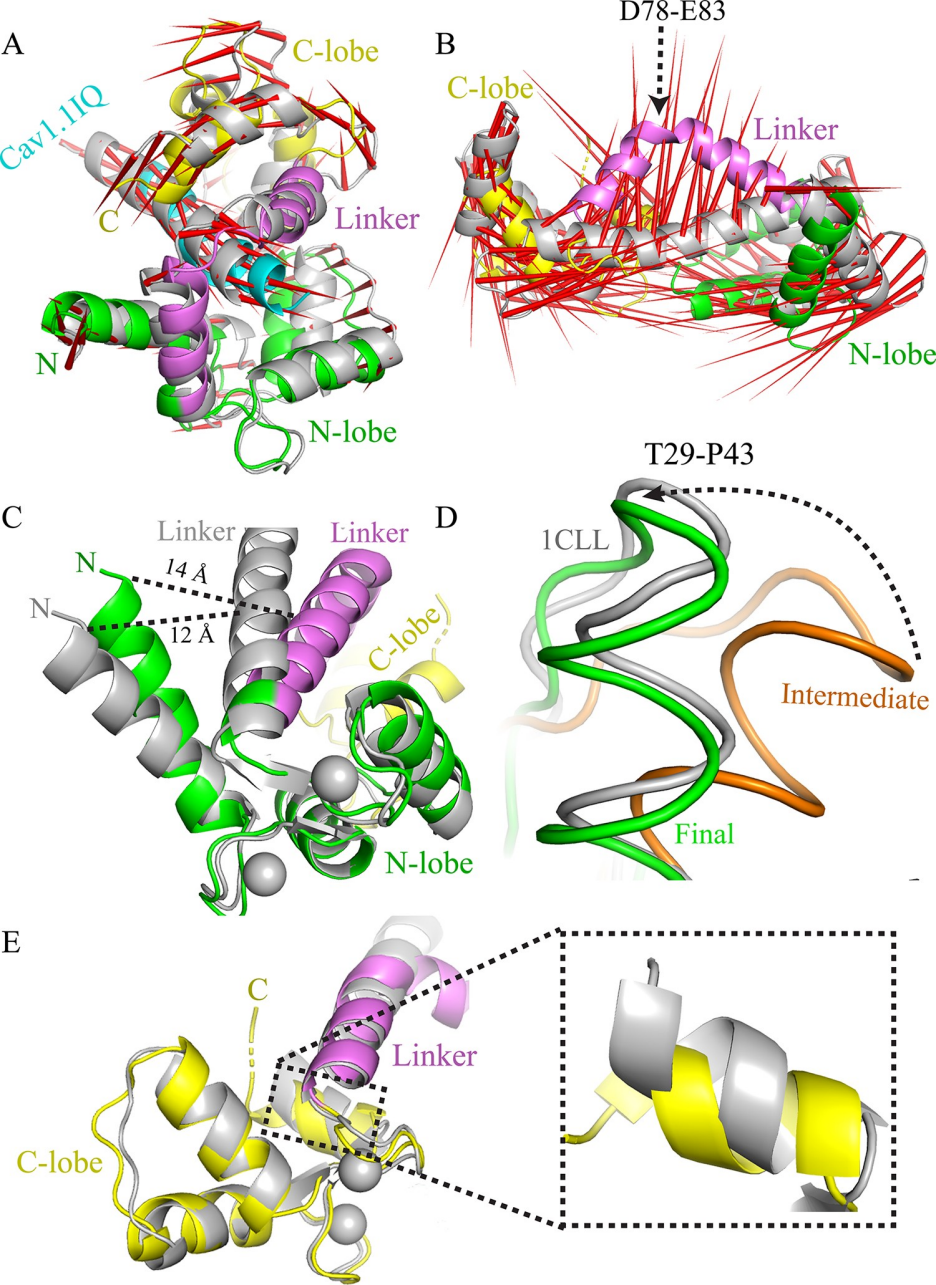
Principal component study of CaM. **(A)** Among various Ca^2+^/CaM-IQ motif complexes, only the Ca^2+^/CaM-Cav1.1IQ structure (2VAY) reveals slight movements in the C-lobe and the IQ peptide. Modevector (red arrow) represent the flexible region. **(B)** Extended Ca^2+^/CaM shows significant conformational change in the central helix region. The central helix bends by ~84° and adopts a compact form. **(C)** Intermediate states of the N-lobes during structural transition of the linker region. The helix α1 (EF1) is seen 2Å away from its initial position. **(D)** Similarly, region T29-P43 is observed in an intermediate position (orange) during the course of simulation. This fragment attains final stable position at the end of simulation (green). **(E)** C-lobe is similar to the original structure except for the α2 helix region of EF4 (inset).

Principal component analysis (PCA) is a standard procedure to analyse the variable correlations from a MD trajectory. PCA can reveal the most important motions in a system. In brief, PCA technique is applied to systematically reduce the number of dimensions so that protein dynamic motions can be filtered from the largest to the smallest spatial scales [58]. We performed PCA to understand the motion of the different lobes of CaM, its linker region, and IQ motifs (S2 Fig). PCA analysis suggests that Ca^2+^/CaM IQ motif complexes are relatively stable and do not undergo significant movement. It seems that in the compact Ca^2+^/CaM IQ complex, the linker has undergone maximum bending. Further bending of the linker is not energetically favourable. However, the degree of bending depends on the target peptide as different orientations are observed in different CaM-IQ complexes [33]. The IQ peptides bind CaM in the most stable way. Only the terminal residue of CaM and IQ motifs exhibited slight movement (S2 Fig). Noticeable movement was observed in CaM/Cav1.1IQ (2VAY). The C-lobe showed highest degree of movement (in anti-clockwise manner) in respect to the N-lobe (Fig 5A). The linker residues (77–82) also showed slight movement.

As mentioned above, bending of the linker depends on the target and the orientation of the two lobes is locked by IQ peptides. Hence, in a particular Ca^2+^/CaM IQ complex, the position of the lobes is fixed in the most stable orientation, and further movement is restricted. Therefore, PCA analysis do not show any domain specific movement despite that the lobes orientation is different in various Ca^2+^/CaM IQ complexes. Whereas target free extended and compact CaM shows flexible lobes and linker region, the target bound Ca^2+^/CaM IQ motif complexes do not undergo any significant movement.

### Structural rearrangements in CaM undergoing transition from extended to compact form

Proteins are highly dynamic in aqueous solution and can adopt multiple conformations required for functioning. CaM is a great example with the ability to assume multiple conformations [60] (Fig 1). PCA extracted trajectory was analysed to understand how CaM transitions to the compact form. The extended CaM (1CLL) undergoes a wide range of conformations in order to reach the compact form (Fig 5B). This starts with the unwinding of the region D78-E83 [43]. Unwinding favours linker bending and that in turn allows the lobes to come closer resulting in the compact form (Fig 5B and S1 Movie). The whole structure transition occurs in a nanosecond [40]. Then, as seen in the S1 Movie and in Fig 5B, both lobes move from their initial positions to adopt the compact form. The central helix bends by ~84° (Fig 5B). We used the coordinates of the final simulated structure and performed DALI server [61] search to find structural homologs. However, no matches to CaM conformations deposited in Protein Data Bank were obtained. The most similar PDB deposited structure was 6OS4 (Z score 12.9 and RMSD 2.7 Å) which is CaM complexed with prenyl moiety of GTPase KRAS4b involved several signal transduction pathways [62]. Thus, the simulation reveals that CaM adopts a unique conformation.

In the N-lobe, the gap between α1 (EF1) helix and the central helix increases (Fig 5C). This likely represents an intermediate state. During the transitioning from the extended to the compact form, the EF-hand helices also transiently rearrange to facilitate the linker bending [39, 40]. After CaM has adopted the compact form, EF-hand helices regain their original positions. It seems that in the current simulated structure, the central linker is bent to the compact form, however, helix α1 (EF1) is yet to reach its final state. Similarly, the region T29-P43 is observed in the transient intermediate state during simulation but attains the final state at the end of the simulation period (Fig 5D). However, no significant structural differences were observed in the C-lobe. Only the terminal residues (140–147) were slightly shifted (Fig 5E). It seems that the C-lobe has already undergone the structural arrangement required for the formation of the compact structure. As discussed earlier, the N- and C-lobes are not identical as they differ in Ca^2+^ affinities and other biophysical properties [63, 64]. Therefore, their folding pathways are not coherent, probably structural rearrangement in C-lobe finishes earlier than N-lobe.

**Fig 6:**
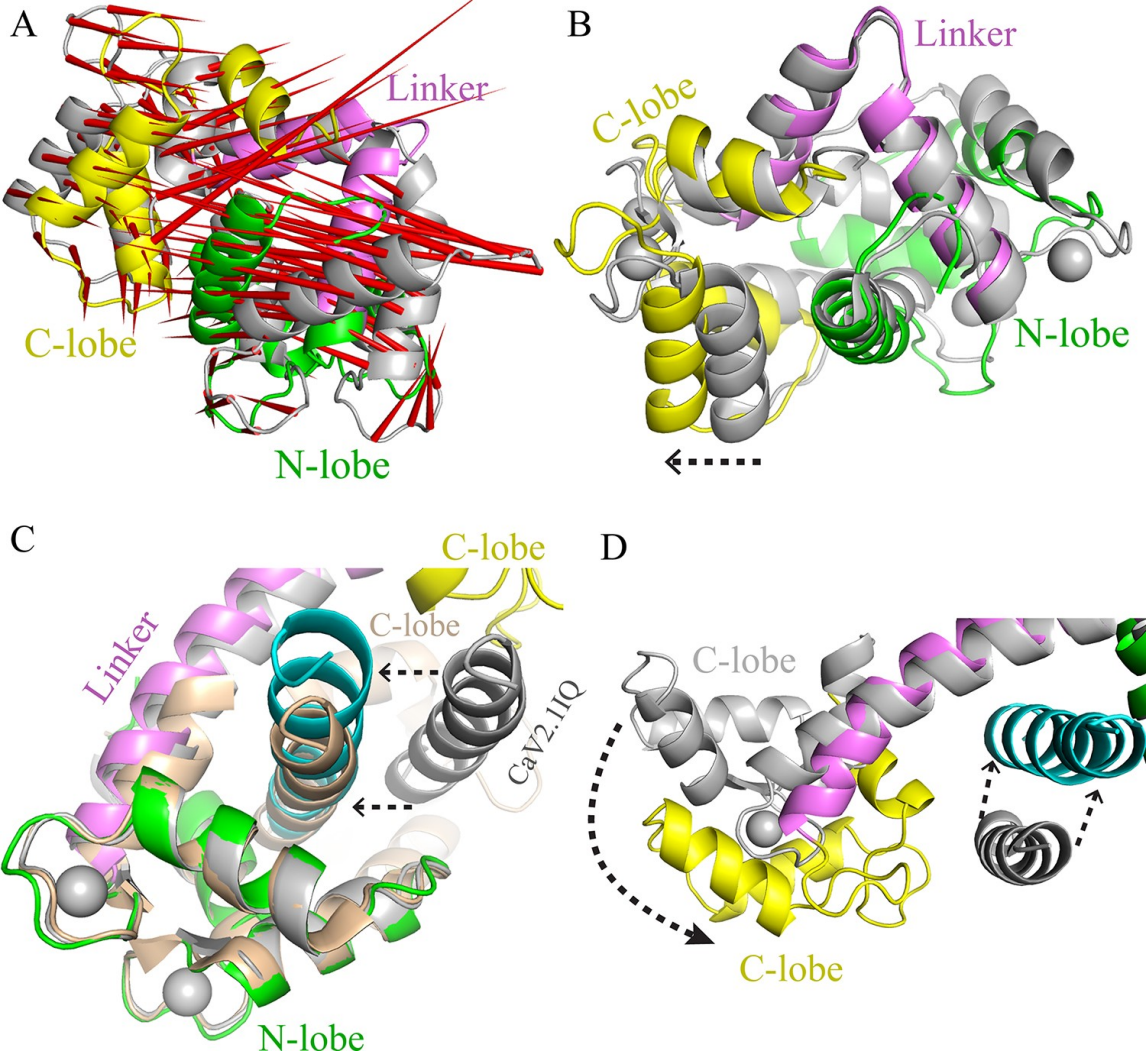
**(A)** Compact Ca^2+^/ CaM is dynamic in solution. Both lobes and the linker are deviate from their initial positions. **(B)** The lobes move apart slightly and widens the compact Ca^2+^/CaM. **(C)** In an artificial complex of CaM and IQ motif (grey), IQ peptide moves towards and binds CaM in its natural binding position. Final simulated structure is show in multicolour (N-lobe; green, C-lobe; yellow, and linker; violet). Ca^2+^/CaM-Cav2.1IQ (3DVM) is shown in wheat colour. **(D)** Following peptide binding to CaM, the central linker bends. The movement of peptide and bending of the linker is shown by dotted arrows.

We also analysed PCA extracted trajectory of compact Ca^2+^/CaM (1PRW). Both the N- and C-lobes move towards each other resulting in a more compact form. However, no apparent differences were observed within the lobes. The EF-hand motifs did not show any movement. Only the N-terminal α1 helix shifted slightly (Fig 6A). However, the linker region deviated significantly from its original positions causing the C-lobe to reorient into a new position (Fig 6B). No conformational changes occurred in the linker and the bending of the linker was similar to the one observed in the crystal structure. However, the N- and C-lobes moved slightly further apart due to the new position of the C-lobe (Fig 6B). Thus, the compact CaM fluctuates in solution and an intermediate state was captured in this study. The C-lobe does not undergo any significant conformational change. Dali server search suggested that the closest structural homologue to the simulated structure is CaM complexed with inhibitor sphingosylphosphorylcholine (PDB ID: 3IF7, Z score 20.1 and RMSD 2.0). Sphingolipid binds in the IQ motif binding site and CaM is in the compact form (S3 Fig).

### Conformational landscape during complex formation

To understand the interaction mechanism between, we prepared an artificial system between extended Ca^2+^/CaM (1CLL) and the IQ motifs (3DVM CaV2.1IQ). The IQ motifs were placed randomly near the residue D80 (CaM) and 15Å (10^−10^ meter) away from the linker without making any contacts with CaM (Fig 6C). MD simulation was carried out with similar biophysical conditions as before. The CaV2.1IQ motif travels towards CaM, finds its binding site, and binds CaM in antiparallel orientation at the same site as in the crystal structure (Fig 6C). Thus, the target peptide can locate its binding site on CaM in the solution. After peptide binding, the central linker starts to bend pivoted at T79 (Fig 6D). Bending of the linker brings the C-lobe close to the peptide and, eventually, the compact form CaM forms. We could see that the linker was starting to bend in our final simulated structure, though it had not yet reached the full extension of the bend (Fig 6D). This finding indicates that during complex formation, first the IQ peptide binds to CaM followed by peptide induced bending of the central helix. It is the target peptide that induces the various degrees of bending of the central helix.

## Discussion

Many enzymes and proteins undergo conformational changes for their activity and adopt multiple conformations. The protein folding, ligand interaction, and protein-protein interactions are very intricate processes that cannot be fully explained by experimental observations. However, the advent of supercomputers and improved MD simulation algorithms enable the visualization of putative folding pathways [65]. MD simulations provide detailed atomic level description of protein dynamics using empirical force fields [66]. MD simulations are uniquely suited for examining the dynamics of protein–protein complexes with atomistic detail. While X-ray crystallography and solution nuclear magnetic resonance (NMR) spectroscopy structures do not address the dynamic interactions between the protein complexes, the fluctuations and conformational changes within biological macromolecules can be studied using MD simulations. Atomic-level MD simulations allow to characterize the dynamics of protein-protein complexes computationally.

The interaction of Ca^2+^ ion with CaM causes significant structural rearrangements. On the other hand, Ca^2+^ ions removal from CaM causes reduced stability [45, 67, 68]. How target protein reduces the binding of Ca^2+^ ions? In apo CaM, EF-hand loop residues are very flexible and can therefore easily coordinate free Ca^2+^ ions. When the target protein binds the CaM, it restricts the flexibility of the EF-hand loop residues thereby reduces the affinity for Ca^2+^ ions. In this work, we show that the flexibility of EF-hand residues directly affects the binding of Ca^2+^ ions. The more flexible EF-hand residue binds Ca^2+^ more strongly (Fig 3). The target proteins regulate the CaM affinity for Ca^2+^ ions and can alter the CaM conformations [69]. Therefore, CaM can sense a broad range of Ca^2+^ concentrations depending on the target it binds. For instance, in the CaM-CaMBD2-b complex, Ca^2+^ binds more stably in N-lobe compared to C-lobe [69].

Furthermore, the binding of target brings the lobes from *trans* to *cis* orientation. In *cis* conformation, linker is more stable, and the two lobes have fixed positions. This is corroborated by lower RMSD values of compact Ca^2+^/CaM and Ca^2+/^CaM/IQ motif complexes (Fig 2). However, the relative orientation of N- and C-lobes also depends on ionic distribution in the aqueous solution. At lower ionic strength, nonspecific ions accumulate near linker. The changes in ion concentration near the protein surface allows N- and C-lobes to sample multiple orientations. The CaM protein is stable at physiological ionic strength. However, decrease in ionic strength or pH favours the compact form [40].

Cooperative conformational changes are seen in many enzymatic reactions. To understand how CaM forms a complex with its target, structural and dynamic information is required at single molecule level. Single-molecule methods (e.g. Single-molecule fluorescence resonance energy transfer (smFRET) and single-molecule force spectroscopy) offer a possibility to track the time dependent conformations of proteins [70, 71]. MD simulation can be also used to study the association and dissociation of two proteins during a single trajectory. Our MD simulations successfully reproduced the Ca^2+^/CaM transition pathway from extended to compact form. Residue E31 is a charged polar amino acid that regulates the CaM transition from extended to compact form. Its ionization state is highly sensitive to pH of the environment. Wild type CaM undergoes transition from extended to compact form in microseconds. However, amino acid mutation E31A results in extended to compact transition within tens of nanoseconds [40]. The VGCC IQ motifs bind CaM in parallel and antiparallel orientation. In the parallel orientation, the N-lobe and the C-lobe interact with the N-terminal and C-terminal of IQ motifs, respectively, whereas in the antiparallel binding vice versa interactions occur. In our simulation, the IQ motif binds CaM in the antiparallel orientation (Fig 6C). Antiparallel binding is reported for CaV2.1IQ motif (aa 1961–1982) in the crystal structure [72]. Interestingly, a slightly shorter CaV2.1IQ motif (1960–1976) binds Ca^2+^/CaM in the parallel orientation [73]. The CaV1.2IQ motif also interacts with Ca^2+^/CaM in parallel orientation [74, 75]. However, most of the single helix peptides bind Ca^2+^/CaM in antiparallel orientation [76]. In parallel orientation, majority of the residues involved in CDF (calcium-dependent facilitation) do not contact the Ca^2+^/C-lobe, which is responsible for CDF [75, 77]. Instead, they interact with Ca^2+^/N-lobe, which is responsible for CDI (calcium-dependent inactivation) [75, 77]. Therefore, the antiparallel orientation of CaV1.2IQ motif complies with the functional data.

## Conclusion

In this study, we report the dynamic states of Ca^2+^/CaM in extended and compact forms. EF-hand helices are highly dynamic in solution in both the apo CaM and the extended Ca^2+^/CaM. In compact Ca^2+^/CaM, many of these helices become stabilized due to structural rearrangements. However, Ca^2+^/CaM and VGCC IQ motif complexes are highly stable in solution with RMSD less than 0.15 nm. We also report the process of the formation of the complex between the Ca^2+^/CaM and the IQ motif. Cav2.1 IQ motif was placed far from CaM in solution, however, it moved towards CaM and forms complex with it. We captured the conformational landscape of CaM undergoing extended to compact form upon binding the IQ peptide.

## Supporting information

S1 Fig In apo CaM and extended Ca^2+^/CaM, the loop 3/4 (N111-E120) between EF3 and EF4 is highly flexible (RMSF ~0.57 nm) (Fig 3A). In apo CaM and extended Ca^2+^/CaM, α1 (EF1) is positioned too far to make any contacts.
(TIF)

S2 Fig The complexes **(A)** Ca^2+^/CaM-Cav1.2IQ, **(B)** Ca^2+^/CaM-Cav2.1IQ, **(C)** Ca^2+^/CaM-Cav2.2IQ, and **(D)** Ca^2+^/CaM-Cav2.3IQ do not show significant movement in solution. Only the central helix reveals a slight shift.
(TIF)

S3 Fig The final simulated compact CaM form reveals structural homology with CaM bound to inhibitor sphingosylphosphorylcholine (SPU). The inhibitor binds to CaM at the conventional IQ motif binding site.
(TIF)

S1 Movie Dominant motion of the extended Ca^2+^/CaM during transitioning from extended to compact form. (AVI)

S2 Movie Dominant motion of the artificial complex between Ca^2+^/CaM and IQ peptide. The peptide moves towards CaM and its lobes start to come closer to one another.
(AVI)
