# Supplementary figures and images for: The mechanism of complex formation between calmodulin and voltage gated calcium channels revealed by molecular dynamics
Source: PLoS One. 2021 Oct 5;16(10):e0258112. doi: 10.1371/journal.pone.0258112 (PMC8491939; doi:10.1371/journal.pone.0258112)

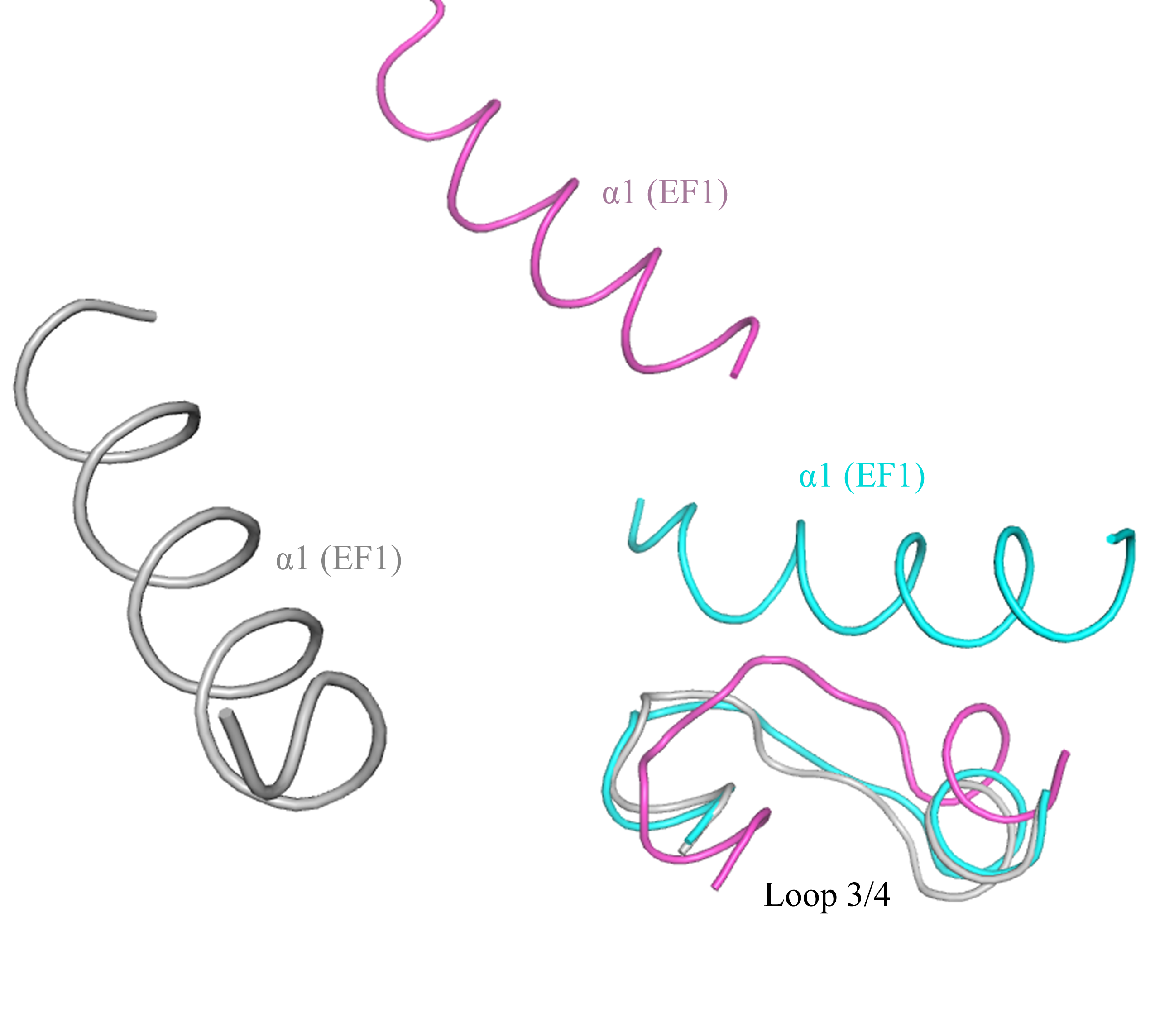

Supplement: S1 Fig — In apo CaM and extended Ca2+/CaM, α1 (EF1) is positioned too far to make any contacts. (TIF) [file pone.0258112.s001.tif]

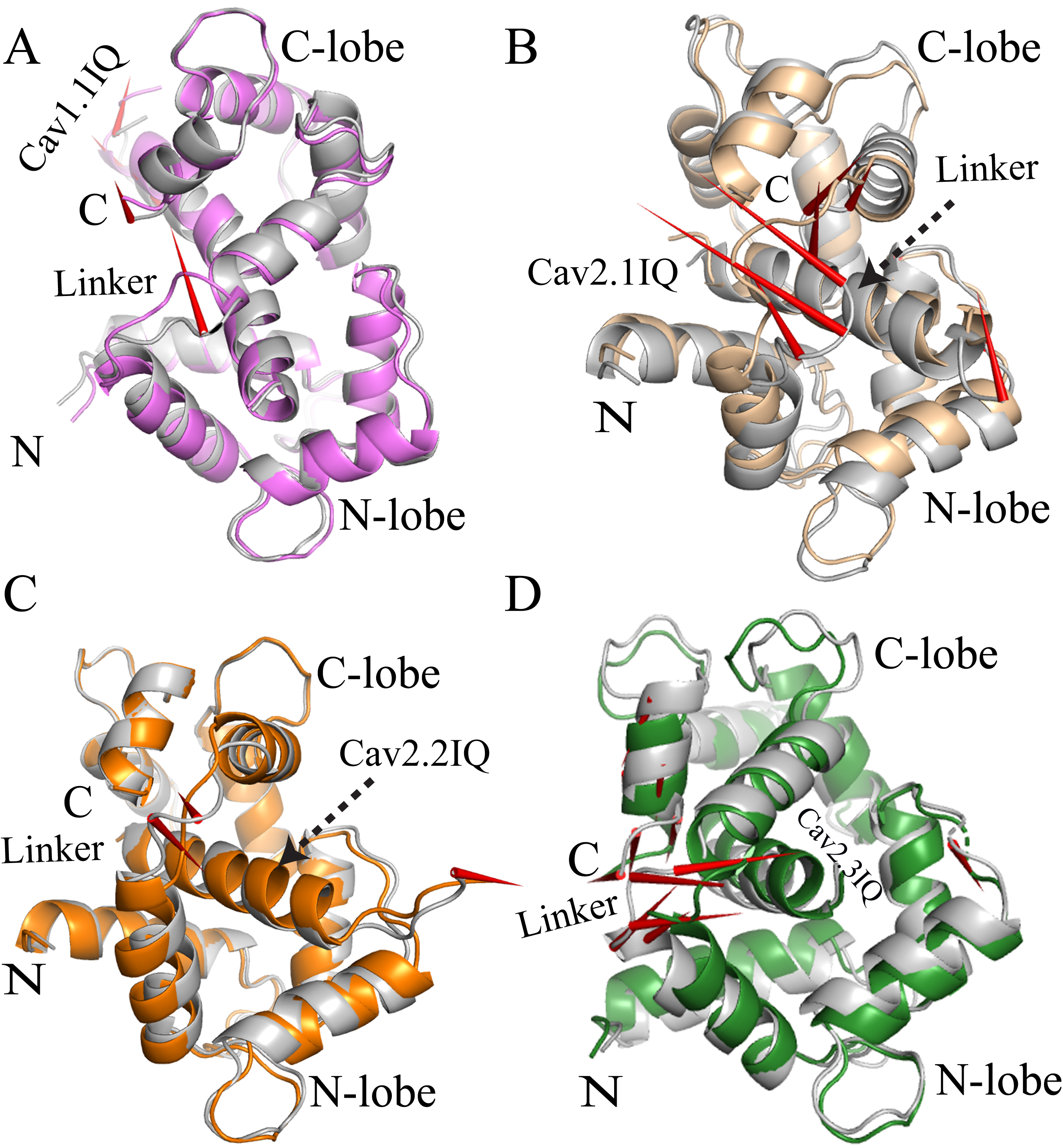

Supplement: S2 Fig — The complexes (A) Ca2+/CaM-Cav1.2IQ, (B) Ca2+/CaM-Cav2.1IQ, (C) Ca2+/CaM-Cav2.2IQ, and (D) Ca2+/CaM-Cav2.3IQ do not show significant movement in solution. Only the central helix reveals a slight shift. (TIF) [file pone.0258112.s002.tif]

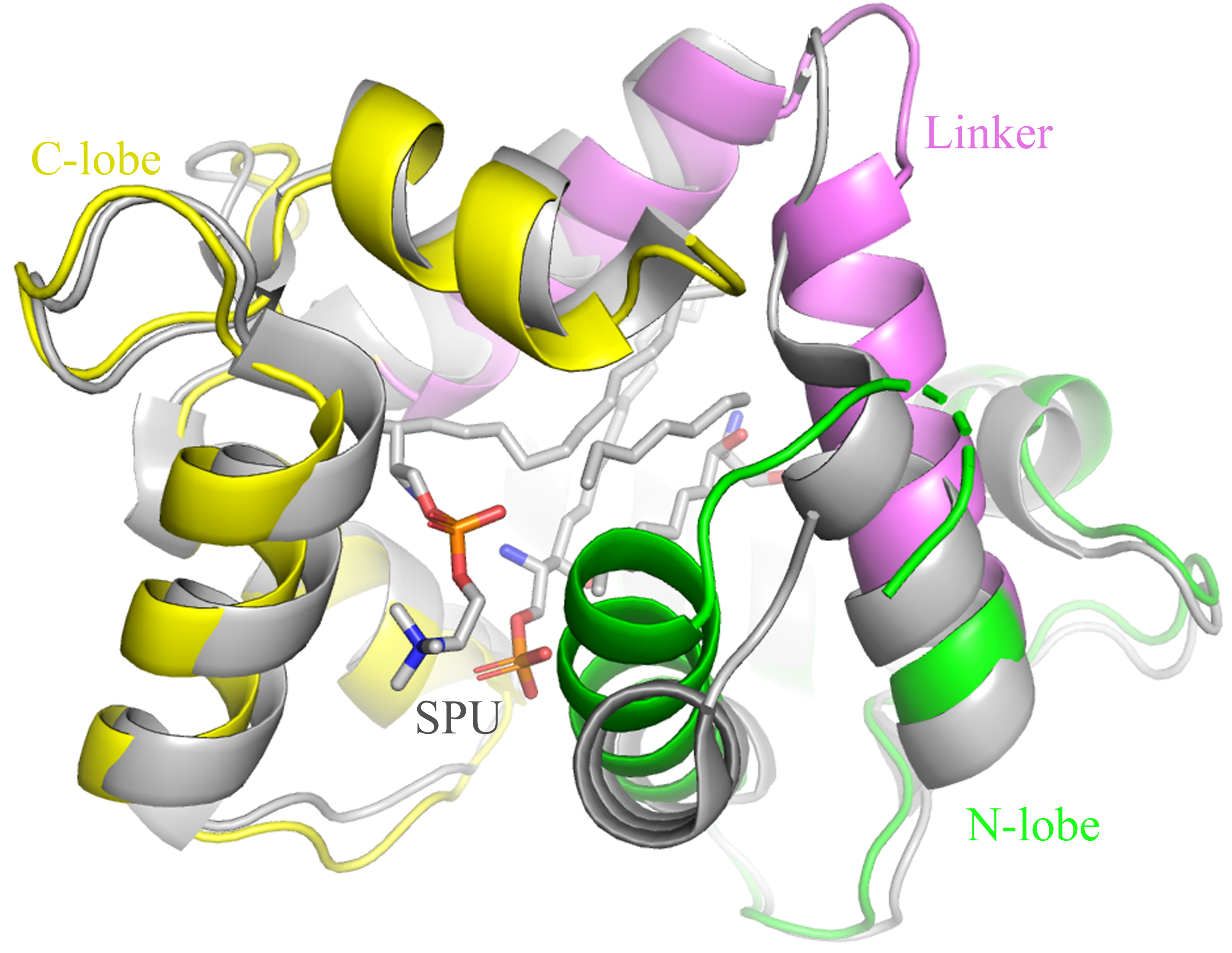

Supplement: S3 Fig — The inhibitor binds to CaM at the conventional IQ motif binding site. (TIF) [file pone.0258112.s003.tif]
